# Supplementary material for: PKD1L1 Is Involved in Congenital Chylothorax
Source: Cells. 2024 Jan 12;13(2):149. doi: 10.3390/cells13020149 (PMC10814685; doi:10.3390/cells13020149)
Supplement: Supplementary file 1 [file cells-13-00149-s001.zip › cells-2733845-supplementary.pdf]

Supplemental Table S1. Molecular Details and Clinical Features of all five index families analysed with ES.

		Family 1 (CHT3)	Family 2 (PUV146)	Family 3 (CHT17)	Family 4 (HRZ2)	Family 5 (HRZ10)
Molecular Details	**Candidate Gene**	PKD1L1	PKD1L1	DNAH5	No Results	No Results
	**Zygosity**	Compound Heterozygous	Compound Heterozygous	Compound Heterozygous		
	**Inheritance**	Autosomal Recessive	Autosomal Recessive	Autosomal Recessive		
	**gDNA location**	Chr7:47944902C>T<br>Chr7:47913548A>T	Chr7:47869647C>A<br>Chr7:47968998delT	Chr5:13850661G>A<br>Chr5:13882811T>A		
	**Variant location**	c.1543G>A, p.Gly515Arg<br>c.3845T>A, p.Val1282Glu	c.6549G>T, p.Gln2183His<br>c.863delA, p.Asn288Thrfs*3	c.5105C>T, p.Ser1702Phe<br>c.3179A>T, p.Lys1060Met		
Clinical Features	**Sex**	Female	Male	Male	Female	Female
	**Age of Onset**	Congenital	Congenital	Congenital	Congenital	Congenital
	**Primary Phenotype**	Chylothorax, left<br>Hydrops fetalis	Hydrothorax, bilateral<br>Hydrops fetalis	Chylothorax, left	Pleural effusion, left	Chylothorax, bilateral
	**Secondary Phenotype**	Persistent pulmonary hypertension, respiratory failure	Severe pulmonary hypoplasia, persistent pulmonary hypertension, cardio-respiratory failure	Pneumothorax, bilateral<br>Lymphoedema	No	Respiratory distress syndrome, cardio-respiratory failure, persistent foramen ovale
	**Prenatal intervention**	3x shunt insertion, thoracocentesis	3x shunt insertion	No	1x shunt insertion	No
